# Surgery Combined with Radiotherapy Improved Survival in Metastatic Esophageal Cancer in a Surveillance Epidemiology and End Results Population-based Study

**DOI:** 10.1038/srep28280

**Published:** 2016-06-21

**Authors:** San-Gang Wu, Wei-Hao Xie, Zhao-Qiang Zhang, Jia-Yuan Sun, Feng-Yan Li, Huan-Xin Lin, Yong Bao, Zhen-Yu He

**Affiliations:** 1 Department of Radiation Oncology, the First Affiliated Hospital of Xiamen University, Xiamen 361003, People’s Republic of China; 2 Department of Radiation Oncology, Sun Yat-sen University Cancer Center, State Key Laboratory of Oncology in South China, Collaborative Innovation Center of Cancer Medicine, Guangzhou 510060, People’s Republic of China; 3 Eye Institute of Xiamen University, Fujian Provincial Key Laboratory of Ophthalmology and Visual Science, Medical College of Xiamen University, Xiamen 361000, People’s Republic of China

## Abstract

This retrospective study used a population-based national registry to determine the impact of local treatment modalities on survival in patients with metastatic esophageal cancer (EC). The Surveillance Epidemiology and End Results (SEER) database was used to identify patients with metastatic EC from 1988 to 2012. A total of 9,125 patients were identified. There were 426 patients underwent primary surgery, 4,786 patients were administered radiotherapy (RT) alone, 847 patients underwent surgery plus RT, and 3,066 patients without any local treatment. Multivariate analysis results indicated that year of diagnosis, age, race, histologic subtype, grade, and local treatment modalities were independent prognostic factors for overall survival (OS). The 5-year OS were 8.4%, 4.5%, 17.5%, and 3.4% in primary surgery, RT only, surgery plus RT, and no local treatment, respectively (*P* < 0.001). Subgroup analyses showed that the impact of RT was mainly reflected by preoperative radiotherapy, as patients received preoperative radiotherapy had significantly better OS than patients who underwent primary surgery alone and postoperative RT, the 5-year OS rates were 24.7%, 6.5%, and 7.8%, respectively, respectively (*P* < 0.001). Surgery plus RT, especially preoperative RT, may improve long-term survival of patients with metastatic EC.

Esophageal cancer (EC) is a highly lethal malignancy, and the incidence is increasing^1^. In 2015, it has been estimated that there would be 16,980 new EC cases and 15,590 deaths in the United States^2^. Approximately 50% of patients had metastases to distant lymph nodes or organs at the initial diagnosis^3^,^4^. The prognosis of metastatic EC is poor, and the 5-year survival rate is lower than 5%^5^. The palliative treatment in metastatic EC depends mainly on patients’ clinical situation with the goal of reducing cancer-related symptoms and extending survival without compromising quality of life. Systemic treatment consists of chemotherapy, targeted therapy, and best supportive care. Local treatment mainly includes feeding tubes, beam radiation, brachytherapy, and endoscopic management techniques such as dilation and stenting^6^,^7^.

Preoperative chemoradiotherapy may significantly increase the radical resection rate and improve survival for advanced esophageal carcinoma^8^, but the value of surgery plus radiotherapy (RT) for metastatic EC has not yet been clarified. RT is not a first-line treatment for metastatic EC, but RT may improve the patients’ symptoms of obstruction^9^. Studies with small sample sizes have shown that local treatments including surgery could prolong survival in metastatic EC^10^,^11^,^12^,^13^. Studies have shown that surgery and/or radiotherapy can improve survival in patients with stage IV malignant tumors^14^,^15^. In this study, we analyzed the metastatic EC using a population-based national registry (Surveillance Epidemiology and End Results, SEER) to determine the impact of local treatment strategies on survival in metastatic EC.

## Results

### Patient characteristics and treatment

**Figure 1:**
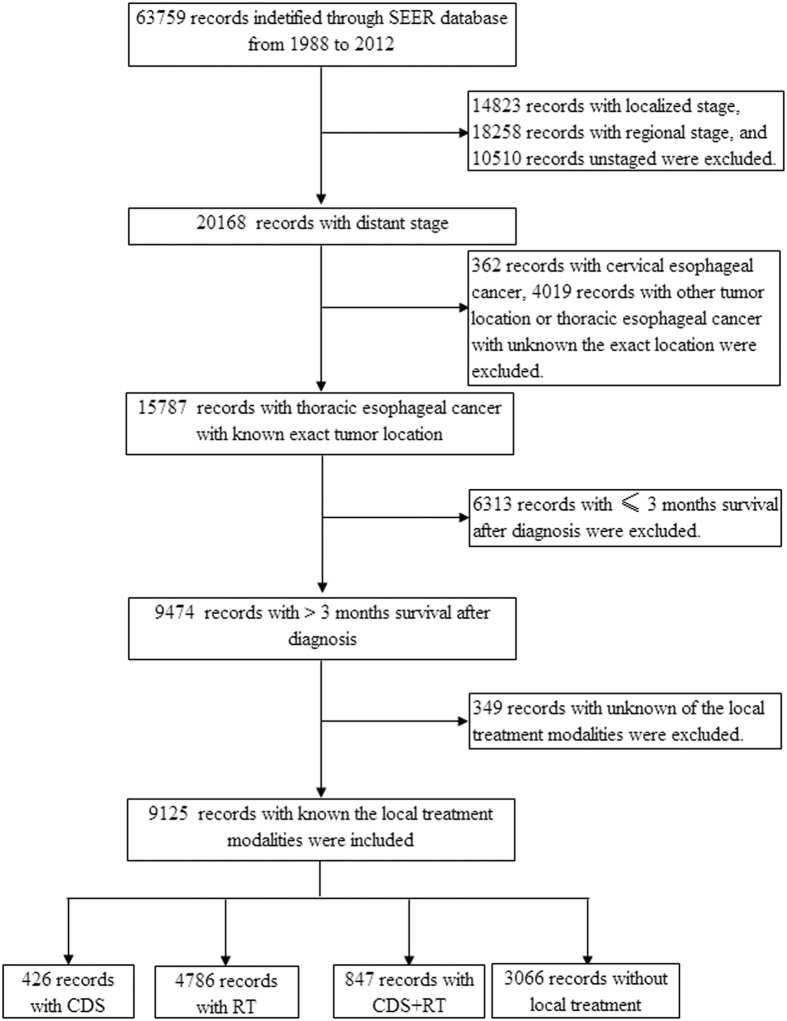
Patients included in analysis.

The SEER database included a total of 63,759 patients with EC in 1988–2012, and 31.6% (20,168 patients) had a distant stage; 9,125 patients met the inclusion criteria of this study (Fig. 1). The patient characteristics are shown in Table 1. The median age of initial diagnosis was 64 years (range, 21–96); 83.5% (7,621/9,125) were white; 82.0% (7,486/9,125) were male; 59.2% (5,406/9,125) had adenocarcinoma; and 76.7% (6,995/9,125) had a lower thoracic esophageal cancer. Local treatment modalities were as follows: 426 (4.7%) patients underwent primary cancer-directed surgery (CDS); 4,786 (52.4%) were primary RT alone; 847 (9.3%) underwent CDS plus RT; and 3,066 (33.6%) were not administered any local treatment. Among patients who underwent CDS plus RT, 57.3% (485/847) were administered preoperative radiotherapy, while 38.3% (324/847) were received postoperative radiotherapy and 4.5% (38/847) were underwent both preoperative and postoperative RT.

### Survival

**Figure 2:**
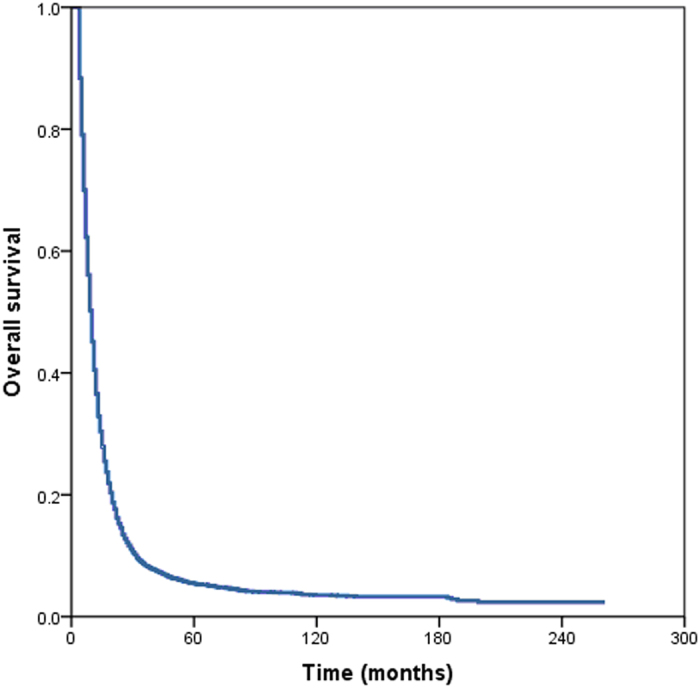
Overall survival of patients with metastatic esophageal cancer.

The median follow-up time for all patients was 9 months (range, 4–261 months) with a median survival time was 10 months. The 1 year, 2 years, 3 years, 5 years, and 10 years OS rates were 40.5%, 14.6%, 8.4%, 5.4%, and 3.5%, respectively (Fig. 2).

### Prognostic factors analysis

Univariate analysis showed that year of diagnosis, age, race, tumor histology, grade, and local treatment modalities were risk factors for OS (Table 2).

Multivariate analysis indicated that year of diagnosis, age at diagnosis, race, tumor histology, grade, and local treatment modalities were independent prognostic factors for OS. Patients who underwent primary CDS was significantly better OS than that of patients who were primary RT alone (HR, 1.440; 95% CI, 1.287–1.611; *P* < 0.001) and who were not received any local treatment (HR, 1.602; 95% CI, 1.427–1.799; *P* < 0.001). Surgery combined with RT could further improve survival (HR, 0.793; 95% CI, 0.693–0.908; *P* = 0.001) (Table 3).

### Survival after local treatment

**Figure 3:**
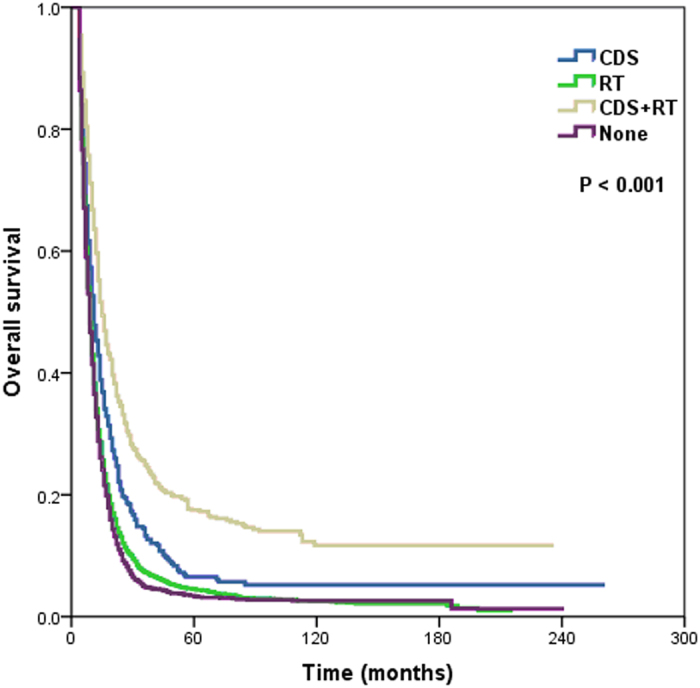
Overall survival of patients with metastatic esophageal cancer undergoing different local treatment modalities (CDS, cancer-directed surgery; RT, radiotherapy).

**Figure 4:**
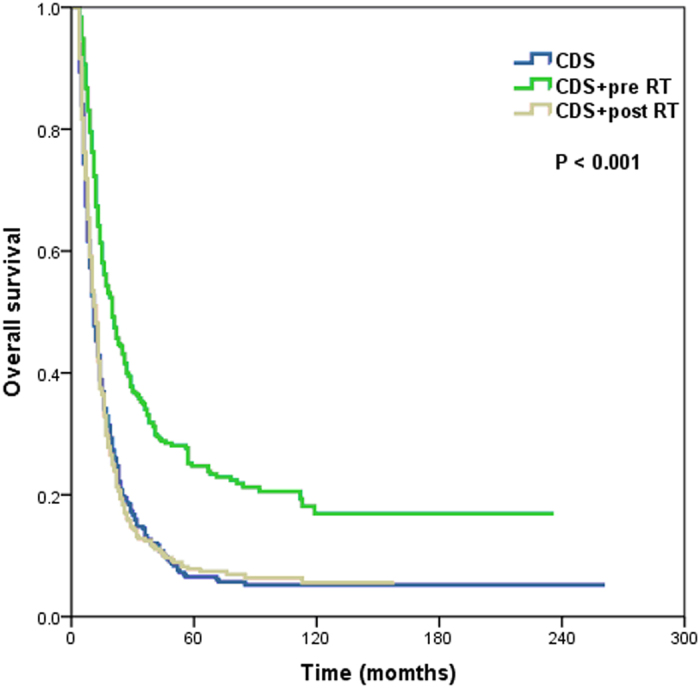
Overall survival of patients with metastatic esophageal cancer undergoing surgery combined with radiotherapy (CDS, cancer-directed surgery; RT, radiotherapy; pre, preoperative; post, postoperative).

The 5-year OS rates were 8.4%, 4.5%, 17.5%, and 3.4% for primary CDS, RT alone, CDS plus RT, and no local treatment, respectively, with a median survival time of 11.0, 9.0, 15.0, and 9.0 months, respectively (*P* < 0.001) (Fig. 3). Patients who were received preoperative RT was significantly better OS than that of patients who underwent primary CDS and CDS plus postoperative RT, with 5-year OS rates of 24.7%, 6.5%, and 7.8%, respectively, and a median survival time of 20.0, 11.0, and 12.0 months, respectively (*P* < 0.001) (Fig. 4).

We determined the effect of local treatment modalities on OS by year of diagnosis. It was significantly associated with OS from 1988 to 1999 (log-rank test *P* < 0.001) (Figure S1A) and from 2000 to 2012 (*P* < 0.001) (Figure S1B). However, the survival benefit was significantly better from 2000 to 2012 for those treated with CDS plus RT.

The prognostic effect of local treatment modalities was examined based on different ethnicities. In black patients, CDS with or without RT improved OS than that of patients who underwent primary RT alone or did not have any local treatment (*P* < 0.001) (Figure S2A). In white patients, OS was better for patients who underwent CDS plus RT, as compared with other local treatment modalities (*P* < 0.001) (Figure S2B).

The effect of local treatment modalities on OS was examined based on sex. In patients who underwent CDS plus RT, OS was significantly better than that of patients who were received other local treatment modalities (*P* < 0.001 for male patients; *P* < 0.001 for female patients) (Figure S3A,S3B). The results were also significant difference in patients who were aged ≤60 years (*P* < 0.001) and aged >60 years (*P* < 0.001) (Figure S4A,S4B).

In addition, CDS plus RT provided the OS benefit in patients with squamous cell carcinoma (*P* < 0.001) (Figure S5A), adenocarcinoma (*P* < 0.001) (Figure S5B), grade I-II (*P* < 0.001) (Figure S6A), and grade III-IV *P* < 0.001) metastatic EC patients. (Figure S6B).

The OS rates were compared based on tumor location for patients who were underwent different local treatment modalities (Figure S7A–S7C). The prognostic effect of local treatment modalities was also found in patients with tumors located in the middle thoracic esophagus (*P* < 0.001) and lower thoracic esophagus (*P* < 0.001). However, in patients with tumors located in the upper thoracic esophagus, local treatment modalities were not associated with OS (*P* = 0.272).

## Discussion

Given the limited of studies with small sample sizes investigating the effect of local treatment in metastatic EC^10^,^11^,^12^,^13^. In this study, we explored the prognostic value of local treatment modalities including CDS and RT in metastatic EC based on 9,125 metastatic EC patients in the SEER database and our results found that surgery plus RT could significantly improve survival in metastatic EC.

Systematic therapy is still the first-line treatment for metastatic EC. The main purpose of local treatment lies in effective control of dysphagia, pain, bleeding, and other symptoms. The potential value of surgery and RT in metastatic EC remains controversial. In a study by Schauer *et al.*, 19 patients with stage IV Barrett’s adenocarcinoma received multimodality therapy including resection of the primary tumor. No significant difference was found in postoperative morbidity and mortality between metastatic EC and locally advanced EC, but the median survival was only 9 months^16^. Tanaka *et al.* also found that surgery did not improve survival in stage IVB EC with distant organ metastasis (*P* = 0.1291)^5^. Wang *et al.* included 96 patients with stage IV EC who were received palliative chemotherapy and concurrent chemoradiotherapy (CRT), of which 14 patients underwent surgery after neoadjuvant therapy and surgery had significantly better survival than those who did not^11^. Two related studies also showed that long-term survival could be achieved after resection of the primary tumor and metastases of stage IV EC^12^,^13^. In our study, 1,273 patients received surgery with or without RT, and surgery combined with RT could significantly improve survival. Thus, multimodality therapy including surgery and RT has the potential to prolong survival in metastatic EC.

Multimodality therapy is the dominant research direction in metastatic EC. Our subgroup analysis showed that in 2000–2012, patients who underwent surgery plus RT obtained a significantly better survival than patients in 1988–1999. Although the SEER data could not reflect specific conditions in patients regarding chemotherapy and targeted therapy, we speculated that it was closely correlated with of the effect of systemic treatment in metastatic EC^17^,^18^,^19^,^20^. Systemic therapy is the primary treatment of metastatic EC, but local treatment including surgery or RT after effective systemic therapy could further reduce the tumor burden. Therefore, we recommend for future prospective studies to investigate the effect of local treatment in metastatic EC.

Our study showed that patients with upper thoracic esophageal cancer did not benefit from local treatment, which might be related to greater difficulties in surgical treatment in upper thoracic esophageal cancer than middle and lower thoracic esophageal cancer. We could not clarify the effect of surgical treatment in upper thoracic metastatic EC, as only 30 patients underwent surgery with or without RT in this study.

In this study, the 5-year OS for preoperative RT plus CDS could reach 24.7%, while no significant difference in survival was seen for primary CDS and CDS plus postoperative RT (5-year OS, 6.5% and 7.8%, respectively), indicating that preoperative neoadjuvant therapy has a greater value in metastatic EC. Our study found that the OS improvement for surgery plus RT was mainly reflected by preoperative RT which could provide the best chance for the complete resection of primary tumors.

There are several limitations in our study. First, inherent biases exist in any retrospective study. Second, due to the limitations of SEER data, we could not obtain related information including chemotherapy, indications for surgery and RT, and range of non-regional lymph node metastases and distant metastases. In addition, patients with distant SEER stage were intended to approximate stage IV in the TNM staging system, and our results also promoted that OS of distant stage in SEER was substantially similar to that of stage IV esophageal carcinoma. Several different extent of disease schemes have been used in the SEER database. Therefore, a potential difference in the two staging systems should be considered. However, the primary strength of this study was the ability to assess the epidemiology, prognostic factors, and local treatment modalities in metastatic EC using a SEER registry. Although retrospective reviews are generally considered inferior to prospective studies, no prospective study design has been performed to assess the clinical value of local treatment in metastatic EC.

In conclusion, surgery plus RT, especially preoperative RT, may improve long-term survival of patients with metastatic EC. A prospective study on metastatic EC should be conducted to investigate the effect of local treatment in metastatic EC. Our findings may play an important role in local treatment considerations in metastatic EC if further confirmed in studies with larger sample sizes.

## Methods and Materials

### Patients

Data were obtained from the current SEER database to identify patients with EC diagnosed in 1988–2012. We obtained permission to access research data files with the reference number 11252-Nov 2014^21^. Patients included in this study had the following criteria: 1) metastatic thoracic esophageal cancer with a known tumor location; and 2) local treatment modalities including cancer-directed surgery (CDS), beam radiotherapy, CDS plus RT, or no local treatment. Metastatic disease was defined as having a distant stage at diagnosis according to the SEER historic stage. Distant stage was defined as a neoplasm that had spread to parts of the body remote from the primary tumor through direct extension, discontinuous metastasis (e.g., implantation or seeding) to distant organs and tissues, or the lymphatic system to distant lymph nodes^21^. Patients were excluded from the analysis if they had an estimated survival of ≤3 months after diagnosis. SEER data did not require informed consent, and this study was approved by the ethics committee of the Sun Yat-sen University Cancer Center.

### Clinicopathological and treatment factors

The following clinicopathological and treatment factors were collected from the SEER database: year of diagnosis, age at diagnosis, race, tumor histology, tumor location, grade, and local treatment modalities. Vital status including cause of death and duration of follow-up was recorded.

### Statistical analysis

The χ^2^ and Fisher’s exact probability tests were used to analyze differences between qualitative data. Univariate and multivariate Cox regression analyses were generated to analyze risk factors for overall survival (OS). Multivariable analyses were performed for factors that were significantly associated with OS in univariate analyses. Survival rates were calculated and plotted using the Kaplan–Meier method and compared using the log-rank test. All data were analyzed using the SPSS statistical software package, version 21.0 (IBM Corporation, Armonk, NY, USA). A *P* value of <0.05 was considered significant.

## Additional Information

**How to cite this article**: Wu, S.-G. *et al.* Surgery Combined with Radiotherapy Improved Survival in Metastatic Esophageal Cancer in a Surveillance Epidemiology and End Results Population-based Study. *Sci. Rep.* **6**, 28280; doi: 10.1038/srep28280 (2016).

## Supplementary Material

Supplementary Information

## Figures and Tables

**Table 1:** Patient characteristics.

Characteristic	n	CDS (%)	RT (%)	CDS + RT (%)	None (%)	*P-*value
Year of diagnosis						
1988–1992	514	67 (15.7)	288 (6.0)	55 (6.5)	104 (3.4)	<0.001
1993–1997	668	68 (16.0)	406 (8.5)	55 (6.5)	139 (4.5)	
1998–2002	1755	111 (26.1)	898 (18.8)	204 (24.1)	542 (17.7)	
2003–2007	2854	102 (23.9)	1489 (31.1)	258 (30.5)	1005 (32.8)	
2008–2012	3334	78 (18.3)	1705 (35.6)	275 (32.5)	1276 (41.6)	
Race						
Black	1016	49 (11.5)	645 (13.5)	59 (7.0)	263 (8.6)	<0.001
White	7621	356 (83.6)	3832 (80.1)	750 (88.5)	2683 (87.5)	
Other	488	21 (4.9)	309 (6.5)	38 (4.5)	120 (3.9)	
Age						
≤60	3515	159 (37.3)	1773 (37.0)	419 (49.5)	1164 (38.0)	<0.001
>60	5610	267 (62.7)	3013 (63.0)	428 (50.5)	1902 (62.0)	
Sex						
Male	7486	349 (81.9)	3867 (80.8)	736 (86.9)	2534 (82.6)	<0.001
Female	1639	77 (18.1)	919 (19.2)	111 (13.1)	532 (17.4)	
Tumor histology						
Squamous	2757	111 (26.1)	1769 (37.0)	207 (24.4)	670 (21.9)	<0.001
Adenocarcinoma	5406	270 (63.4)	2574 (53.8)	543 (64.1)	2019 (65.9)	
Other	962	45 (10.6)	443 (9.3)	97 (11.5)	377 (12.3)	
Tumor location						
Upper thoracic	483	8 (1.9)	340 (7.1)	27 (3.2)	108 (3.5)	<0.001
Middle thoracic	1647	62 (14.6)	1029 (21.5)	110 (13.0)	446 (14.5)	
Lower thoracic	6995	356 (83.6)	3417 (71.4)	710 (83.8)	2512 (81.9)	
Grade (n = 7653)						
G1	270	14 (3.6)	143 (3.6)	24 (3.2)	89 (3.5)	0.271
G2	2826	138 (35.8)	1522 (38.0)	287 (38.4)	879 (34.9)	
G3-4	4557	234 (60.6)	2339 (58.4)	436 (58.4)	1548 (61.5)	

CDS, cancer-directed surgery; RT, radiotherapy; G1, well differentiated; G2, moderately differentiated; G3, poorly differentiated; G4, undifferentiated.

**Table 2:** Univariate analysis of overall survival.

Characteristic	HR	95% CI	*P-*value
Year of diagnosis	0.975	0.971–0.979	<0.001
Age	1.008	1.006–1.010	<0.001
Race			
Black	1		
White	0.922	0.860–0.988	0.021
Other	0.89	0.793–0.998	0.047
Sex			
Male	1		
Female	0.974	0.920–1.031	0.359
Tumor histology			
Squamous	1		
Adenocarcinoma	0.977	0.931–1.026	0.347
Other	1.092	1.010–1.180	0.027
Tumor location			
Upper thoracic	1		
Middle thoracic	1.061	0.953–1.181	0.280
Lower thoracic	0.982	0.890–1.083	0.716
Grade			
G1	1		
G2	1.028	0.900–1.174	0.684
G3-4	1.179	1.035–1.344	0.013
Local treatment modalities			
CDS	1		
RT	1.291	1.162–1.435	<0.001
CDS + RT	0.690	0.608–0.784	<0.001
None	1.384	1.242–1.542	<0.001

CDS, cancer-directed surgery; RT, radiotherapy; G1, well differentiated; G2, moderately differentiated; G3, poorly differentiated; G4, undifferentiated; HR, hazard ratio; CI, confidence interval.

**Table 3:** Multivariate analyses of overall survival.

Characteristic	HR	95% CI	*P-*value
Year of diagnosis	0.971	0.967–0.975	<0.001
Age	1.007	1.005–1.009	<0.001
Race			
Black	1		
White	0.906	0.834–0.985	0.021
Other	0.884	0.779–1.003	0.055
Tumor histology			
Squamous	1		
Adenocarcinoma	1.099	1.035–1.167	0.002
Other	1.174	1.072–1.286	0.001
Grade			
G1	1		
G2	1.067	0.934–0.985	0.337
G3-4	1.202	1.055–1.371	0.006
Local treatment modalities			
CDS	1		
RT	1.440	1.287–1.611	<0.001
CDS + RT	0.793	0.693–0.908	0.001
None	1.602	1.427–1.799	<0.001

CDS, cancer-directed surgery; RT, radiotherapy; G1, well differentiated; G2, moderately differentiated; G3, poorly differentiated; G4, undifferentiated; HR, hazard ratio; CI, confidence interval.
