# Supplementary material for: Surgery Combined with Radiotherapy Improved Survival in Metastatic Esophageal Cancer in a Surveillance Epidemiology and End Results Population-based Study
Source: Sci Rep. 2016 Jun 21;6:28280. doi: 10.1038/srep28280 (PMC4915008; doi:10.1038/srep28280)
Supplement: Supplementary Information [file srep28280-s1.pdf]

SUPPLEMENTARY INFORMATION

Surgery Combined with Radiotherapy Improved Survival in Metastatic Esophageal Cancer in a Surveillance Epidemiology and End Results Population-based Study

San-Gang Wu [1,*], Wei-Hao Xie[2,*], Zhao-Qiang Zhang[3,*], Jia-Yuan Sun[2], Feng-Yan Li[2], Huan-Xin Lin[2], Yong-Bao[2], Zhen-Yu He[2]

[1] Department of Radiation Oncology, the First Affiliated Hospital of Xiamen University, Xiamen 361003, People's Republic of China

[2] Department of Radiation Oncology, Sun Yat-sen University Cancer Center, State Key Laboratory of Oncology in South China, Collaborative Innovation Center of Cancer Medicine, Guangzhou 510060, People's Republic of China

[3] Eye Institute of Xiamen University, Fujian Provincial Key Laboratory of Ophthalmology and Visual Science, Medical College of Xiamen University, Xiamen 361000, People's Republic of China

* San-Gang Wu, Wei-Hao Xie, and Zhao-Qiang Zhang contributed equally to this work.

Corresponding author:

Yong Bao

Department of Radiation Oncology, Sun Yat-sen University Cancer Center, State Key

Laboratory of Oncology in South China, Collaborative Innovation Center of Cancer Medicine, 651 Dongfeng Road East, Guangzhou 510060, People’s Republic of China; Tel: +86 20 87343543; Fax: +86 20 87343392 E-mail. baoyong@sysucc.org.cn

Zhen-Yu He,

Department of Radiation Oncology, Sun Yat-sen University Cancer Center, State Key Laboratory of Oncology in South China, Collaborative Innovation Center of Cancer Medicine, 651 Dongfeng Road East, Guangzhou 510060, People’s Republic of China; Tel: +86 20 87343543; Fax: +86 20 87343392 E-mail. hezhy@sysucc.org.cn

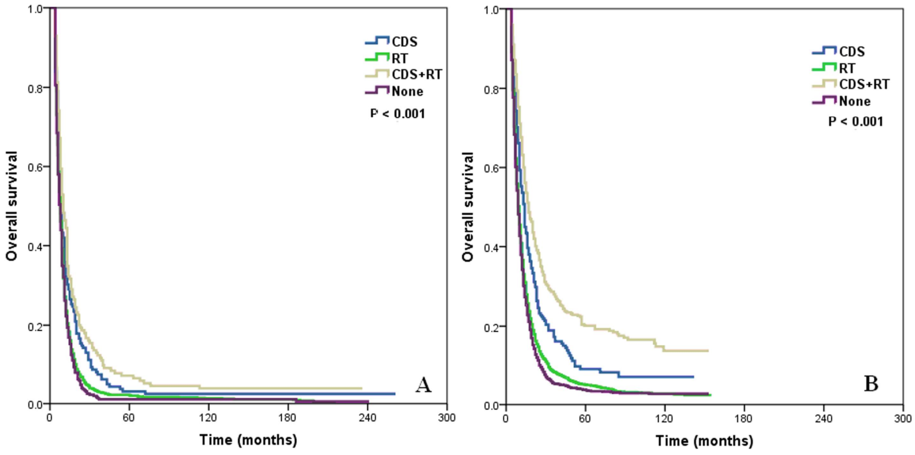

Figure S1. Overall survival of patients with metastatic esophageal cancer undergoing different local treatment modalities by year of diagnosis (A, years 1988-1999; B, years 2000-2012) (CDS, cancer-directed surgery; RT, radiotherapy).

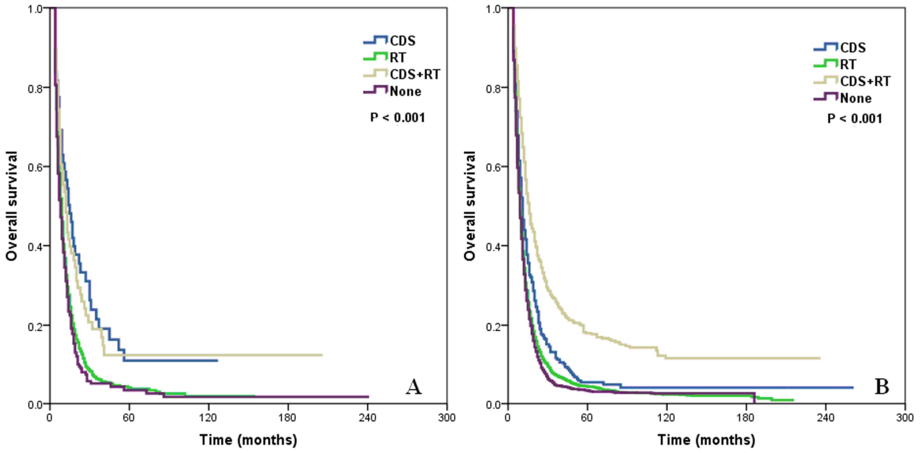

Figure S2. Overall survival of patients with metastatic esophageal cancer undergoing different local treatment modalities by ethnicity (A, black patients; B, white patients) (CDS, cancer-directed surgery; RT, radiotherapy).

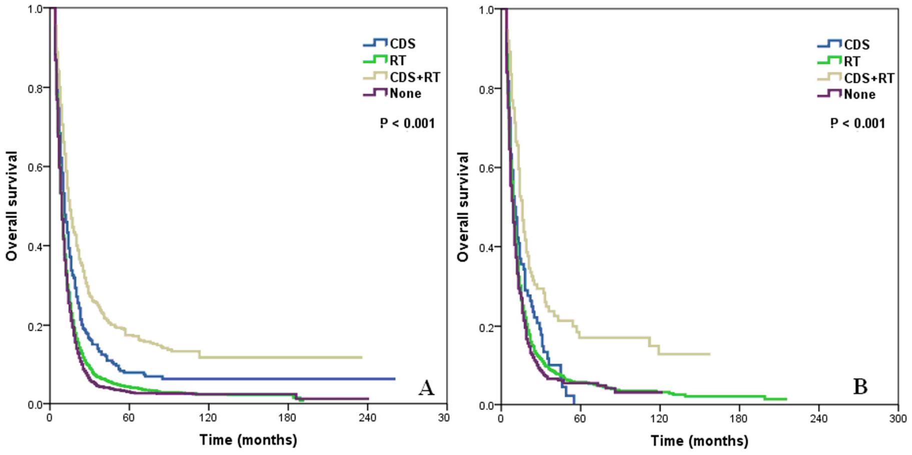

Figure S3. Overall survival of patients with metastatic esophageal cancer undergoing different local treatment modalities by sex (A, male; B, female) (CDS, cancer-directed surgery; RT, radiotherapy).

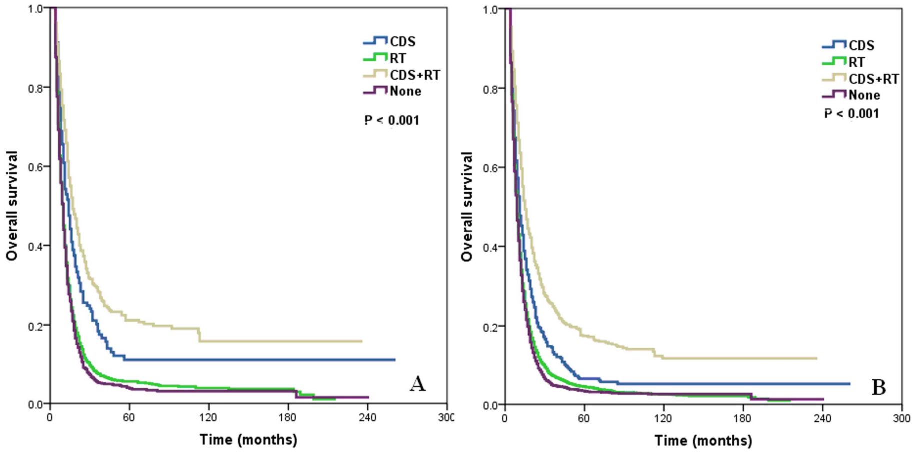

Figure S4. Overall survival of patients with metastatic esophageal cancer undergoing different local treatment modalities by age (A, aged ≤60 years; B, aged >60 years) (CDS, cancer-directed surgery; RT, radiotherapy).

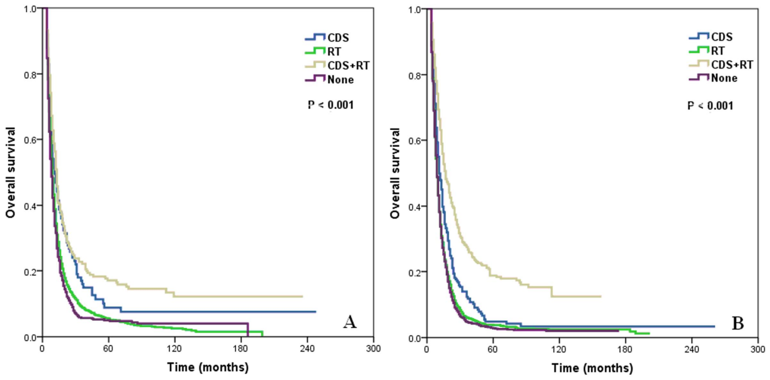

Figure S5. Overall survival of patients with metastatic esophageal cancer undergoing different local treatment modalities by tumor histology (A, squamous cell carcinomas; B, adenocarcinoma) (CDS, cancer-directed surgery; RT, radiotherapy).

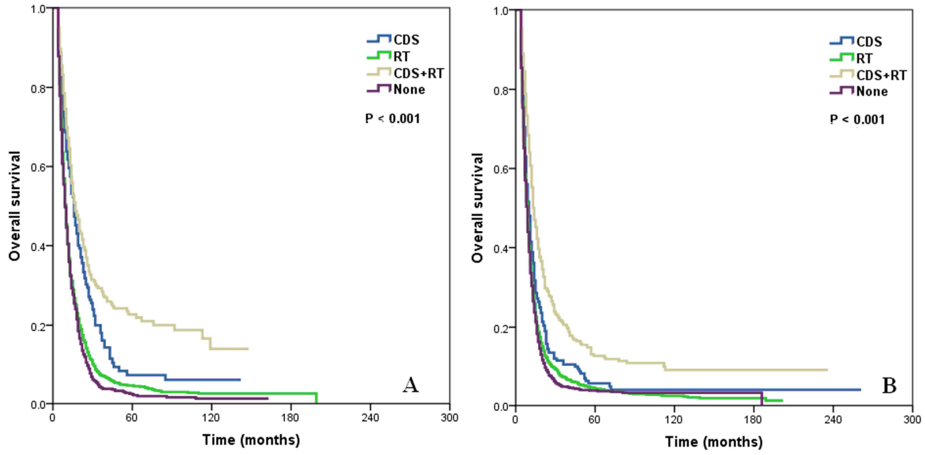

Figure S6. Overall survival of patients with metastatic esophageal cancer undergoing different local treatment modalities by tumor grade (A, well/moderately differentiated; B, poorly/undifferentiated differentiated) (CDS, cancer-directed surgery; RT, radiotherapy).

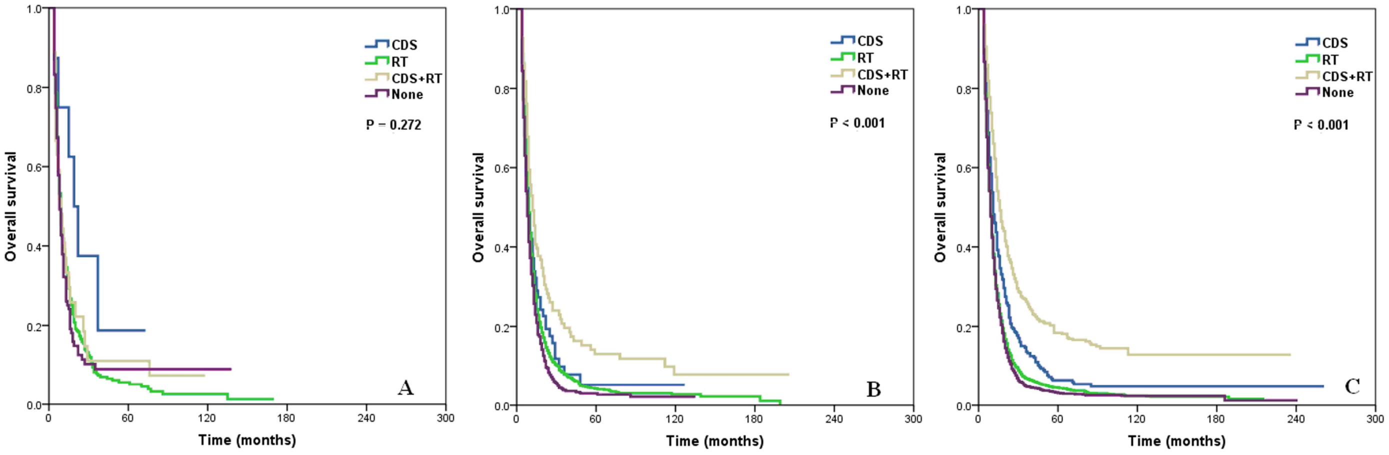

Figure S7. Overall survival of patients with metastatic esophageal cancer undergoing different local treatment modalities by tumor location (A, upper thoracic; B, middle thoracic; C, lower thoracic) (CDS, cancer-directed surgery; RT, radiotherapy).
